# 2,2′-Ethyl­enediisoquinolinium dibromide dihydrate

**DOI:** 10.1107/S1600536809045036

**Published:** 2009-10-31

**Authors:** Jiang-Sheng Li, Peng-Yu Li

**Affiliations:** a School of Chemistry and Biological Engineering, Changsha University of Science & Technology, Changsha 410004, People’s Republic of China

## Abstract

In the title compound, C_20_H_18_N_2_^2+^·2Br^−^·2H_2_O, the complete dication is generated by a crystallographic centre of symmetry. In the crystal, O—H⋯Br, C—H⋯Br and C—H⋯O hydrogen bonds and π–π stacking [shortest centroid–centroid separation = 3.657 (2) Å] help to establish the packing.

## Related literature

For background to supra­molecular chemistry related to the title compound, see: Loeb & Wisner (1998 ▶); Li (2007 ▶). For related structures, see: Li *et al.* (2008 ▶); Xu *et al.* (2007 ▶); Fan *et al.* (2007 ▶).
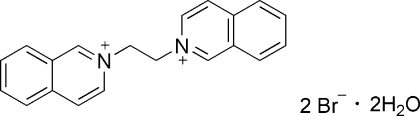


## Experimental

### 

#### Crystal data

C_20_H_18_N_2_^2+^·2Br^−^·2H_2_O
*M* *_r_* = 482.22
Triclinic, 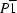

*a* = 7.5203 (15) Å
*b* = 8.0749 (16) Å
*c* = 9.2059 (18) Å
α = 110.34 (3)°
β = 106.96 (3)°
γ = 97.26 (3)°
*V* = 484.9 (2) Å^3^
*Z* = 1
Mo *K*α radiation
μ = 4.20 mm^−1^
*T* = 113 K
0.18 × 0.16 × 0.14 mm

#### Data collection

Rigaku Saturn CCD area-detector diffractometer
Absorption correction: multi-scan (*CrystalClear*; Rigaku, 2005 ▶) *T*_min_ = 0.519, *T*_max_ = 0.591
3994 measured reflections
2262 independent reflections
1800 reflections with *I* > 2σ(*I*)
*R*_int_ = 0.027

#### Refinement

*R*[*F* ^2^ > 2σ(*F* ^2^)] = 0.029
*wR*(*F* ^2^) = 0.078
*S* = 1.07
2262 reflections
126 parameters
H atoms treated by a mixture of independent and constrained refinement
Δρ_max_ = 0.40 e Å^−3^
Δρ_min_ = −0.60 e Å^−3^

### 

Data collection: *CrystalClear* (Rigaku, 2005 ▶); cell refinement: *CrystalClear*; data reduction: *CrystalClear*; program(s) used to solve structure: *SHELXS97* (Sheldrick, 2008 ▶); program(s) used to refine structure: *SHELXL97* (Sheldrick, 2008 ▶); molecular graphics: *SHELXTL* (Sheldrick, 2008 ▶); software used to prepare material for publication: *SHELXL97*.

## Supplementary Material

Crystal structure: contains datablocks global, I. DOI: 10.1107/S1600536809045036/hb5199sup1.cif

Structure factors: contains datablocks I. DOI: 10.1107/S1600536809045036/hb5199Isup2.hkl

Additional supplementary materials:  crystallographic information; 3D view; checkCIF report

## Figures and Tables

**Table 1:** Hydrogen-bond geometry (Å, °)

*D*—H⋯*A*	*D*—H	H⋯*A*	*D*⋯*A*	*D*—H⋯*A*
O1—H1*A*⋯Br1^i^	0.90 (4)	2.41 (4)	3.308 (3)	176 (3)
O1—H1*B*⋯Br1^ii^	0.81 (5)	2.51 (5)	3.313 (3)	178 (5)
C1—H1⋯Br1^iii^	0.95	2.84	3.593 (3)	137
C9—H9⋯Br1^iv^	0.95	2.81	3.691 (3)	154
C10—H10*B*⋯Br1^iv^	0.99	2.87	3.683 (3)	140
C3—H3⋯O1^v^	0.95	2.57	3.396 (4)	145
C4—H4⋯O1^vi^	0.95	2.54	3.380 (4)	147
C10—H10*A*⋯O1^iii^	0.99	2.27	3.214 (4)	158

Symmetry codes: (i) 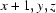; (ii) 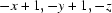; (iii) 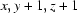; (iv) 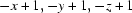; (v) 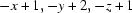; (vi) 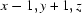.

**Fig. 1.**
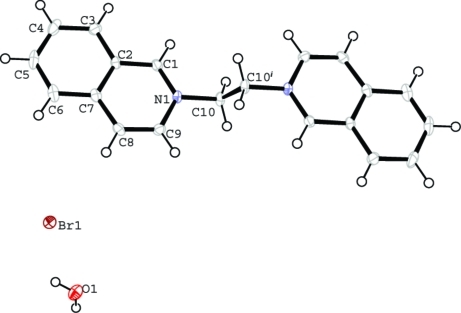
The molecular structure of (I) showing 50% probability displacement displacement ellipsoids. [Symmetry codes: (i) 1 - x, 2 - y, 2 - z.]

## supplementary crystallographic information

### Comment

As part of our ongoing studies of analogs of 1,2-bis(pyridinium) ethane dications (Li *et al.*, 2008), we synthesized a new dication 1,2-bis(isoquinolinium)ethane. Herein, its crystal structure is reported.

The molecular structure of (I) is shown in Fig. 1. The molecule has a centre of symmetry at the mid-point of the C10—C10A bond. The two isoquinoline rings are parallel to each other. The N^+^···N^+^ distance in the title compound is 3.7609 (8) Å, similar to the value previously reported (*ca* 3.75 Å) in the 1,2-bis(pyridinium)ethane dication (Loeb & Wisner, 1998). The crystal structure is stabilized by a series of intermolecular hydrogen bonds (Table 1). The hydrate tends to form an extensive network in the crystal by the aid of Br anions and water molecules. Also, the title cation were stacked *via*π-π interactions between isoquinolinium rings.

### Experimental

The title compound was obtained according to the method of Loeb and Wisner (1998). Light yellow blocks of (I) were grown from its aqueous solution.

### Refinement

The water H atoms were positioned geometrically to acheive a reasonable hydrogen-bonding scheme. The other H atoms were positioned geometrically, with C—H = 0.95 Å for aromatic H and 0.99 Å for methyl H, and were constrained to ride on their parent atoms, with *U*_iso_(H) =1.2*U*_eq_(C).

### Crystal data

**Table:**

C_20_H_18_N_2_^2+^·2Br^−^·2H_2_O	*Z* = 1
*M**_r_* = 482.22	*F*(000) = 242
Triclinic, *P*1	*D*_x_ = 1.651 Mg m^−^^3^
Hall symbol: -P 1	Mo *K*α radiation, λ = 0.71073 Å
*a* = 7.5203 (15) Å	Cell parameters from 1667 reflections
*b* = 8.0749 (16) Å	θ = 2.5–27.9°
*c* = 9.2059 (18) Å	µ = 4.20 mm^−^^1^
α = 110.34 (3)°	*T* = 113 K
β = 106.96 (3)°	Block, light yellow
γ = 97.26 (3)°	0.18 × 0.16 × 0.14 mm
*V* = 484.9 (2) Å^3^	

### Data collection

**Table:**

Rigaku Saturn CCD area-detector diffractometer	2262 independent reflections
Radiation source: rotating anode	1800 reflections with *I* > 2σ(*I*)
confocal	*R*_int_ = 0.027
Detector resolution: 7.31 pixels mm^-1^	θ_max_ = 27.9°, θ_min_ = 2.5°
ω and φ scans	*h* = −8→9
Absorption correction: multi-scan (*CrystalClear*; Rigaku, 2005)	*k* = −8→10
*T*_min_ = 0.519, *T*_max_ = 0.591	*l* = −11→12
3994 measured reflections	

### Refinement

**Table:**

Refinement on *F*^2^	Primary atom site location: structure-invariant direct methods
Least-squares matrix: full	Secondary atom site location: difference Fourier map
*R*[*F*^2^ > 2σ(*F*^2^)] = 0.029	Hydrogen site location: inferred from neighbouring sites
*wR*(*F*^2^) = 0.078	H atoms treated by a mixture of independent and constrained refinement
*S* = 1.07	*w* = 1/[σ^2^(*F*_o_^2^) + (0.0391*P*)^2^] where *P* = (*F*_o_^2^ + 2*F*_c_^2^)/3
2262 reflections	(Δ/σ)_max_ = 0.001
126 parameters	Δρ_max_ = 0.40 e Å^−^^3^
0 restraints	Δρ_min_ = −0.60 e Å^−^^3^

### Special details

**Table:**

Geometry. All e.s.d.'s (except the e.s.d. in the dihedral angle between two l.s. planes) are estimated using the full covariance matrix. The cell e.s.d.'s are taken into account individually in the estimation of e.s.d.'s in distances, angles and torsion angles; correlations between e.s.d.'s in cell parameters are only used when they are defined by crystal symmetry. An approximate (isotropic) treatment of cell e.s.d.'s is used for estimating e.s.d.'s involving l.s. planes.
Refinement. Refinement of *F*^2^ against ALL reflections. The weighted *R*-factor *wR* and goodness of fit *S* are based on *F*^2^, conventional *R*-factors *R* are based on *F*, with *F* set to zero for negative *F*^2^. The threshold expression of *F*^2^ > σ(*F*^2^) is used only for calculating *R*-factors(gt) *etc*. and is not relevant to the choice of reflections for refinement. *R*-factors based on *F*^2^ are statistically about twice as large as those based on *F*, and *R*- factors based on ALL data will be even larger.

### Fractional atomic coordinates and isotropic or equivalent isotropic displacement parameters (Å^2^)

**Table:**

	*x*	*y*	*z*	*U*_iso_*/*U*_eq_	
Br1	0.30313 (4)	0.40880 (4)	0.11468 (3)	0.02152 (11)	
N1	0.4507 (3)	0.9986 (3)	0.7872 (2)	0.0144 (5)	
C1	0.3923 (4)	1.1342 (4)	0.7547 (3)	0.0158 (5)	
H1	0.4226	1.2506	0.8429	0.019*	
C2	0.2857 (4)	1.1081 (4)	0.5912 (3)	0.0148 (5)	
C3	0.2230 (4)	1.2538 (4)	0.5580 (3)	0.0211 (6)	
H3	0.2509	1.3701	0.6457	0.025*	
C4	0.1210 (4)	1.2239 (4)	0.3969 (4)	0.0263 (7)	
H4	0.0762	1.3196	0.3727	0.032*	
C5	0.0829 (4)	1.0516 (5)	0.2675 (3)	0.0260 (7)	
H5	0.0148	1.0340	0.1564	0.031*	
C6	0.1411 (4)	0.9094 (4)	0.2972 (3)	0.0226 (6)	
H6	0.1120	0.7941	0.2078	0.027*	
C7	0.2446 (4)	0.9341 (4)	0.4610 (3)	0.0159 (5)	
C8	0.3097 (4)	0.7935 (4)	0.5028 (3)	0.0182 (6)	
H8	0.2827	0.6753	0.4180	0.022*	
C9	0.4100 (4)	0.8262 (4)	0.6625 (3)	0.0174 (6)	
H9	0.4525	0.7310	0.6893	0.021*	
C10	0.5660 (4)	1.0297 (4)	0.9599 (3)	0.0173 (6)	
H10A	0.6328	1.1608	1.0248	0.021*	
H10B	0.6643	0.9589	0.9589	0.021*	
O1	0.8807 (4)	0.4163 (3)	0.1546 (3)	0.0267 (5)	
H1A	0.994 (5)	0.408 (4)	0.142 (4)	0.022 (9)*	
H1B	0.838 (6)	0.458 (6)	0.088 (5)	0.067 (15)*	

### Atomic displacement parameters (Å^2^)

**Table:**

	*U*^11^	*U*^22^	*U*^33^	*U*^12^	*U*^13^	*U*^23^
Br1	0.02605 (18)	0.02344 (16)	0.01838 (16)	0.01248 (12)	0.00851 (12)	0.00973 (12)
N1	0.0142 (11)	0.0168 (11)	0.0109 (10)	0.0027 (9)	0.0026 (9)	0.0062 (9)
C1	0.0194 (14)	0.0150 (13)	0.0136 (12)	0.0039 (11)	0.0073 (11)	0.0056 (10)
C2	0.0160 (13)	0.0176 (13)	0.0138 (12)	0.0027 (11)	0.0079 (11)	0.0083 (11)
C3	0.0245 (15)	0.0259 (15)	0.0236 (14)	0.0127 (13)	0.0146 (12)	0.0152 (13)
C4	0.0251 (16)	0.0406 (19)	0.0315 (16)	0.0139 (14)	0.0149 (14)	0.0297 (15)
C5	0.0162 (15)	0.0469 (19)	0.0175 (14)	0.0038 (14)	0.0034 (12)	0.0197 (14)
C6	0.0177 (15)	0.0316 (16)	0.0142 (13)	−0.0020 (13)	0.0041 (12)	0.0085 (12)
C7	0.0128 (13)	0.0211 (14)	0.0148 (12)	0.0016 (11)	0.0073 (11)	0.0074 (11)
C8	0.0210 (15)	0.0142 (13)	0.0145 (12)	0.0013 (11)	0.0060 (11)	0.0014 (11)
C9	0.0183 (14)	0.0150 (13)	0.0188 (13)	0.0052 (11)	0.0057 (11)	0.0073 (11)
C10	0.0177 (14)	0.0186 (14)	0.0109 (12)	0.0006 (11)	0.0004 (11)	0.0059 (11)
O1	0.0271 (13)	0.0288 (12)	0.0299 (11)	0.0089 (10)	0.0099 (10)	0.0182 (10)

### Geometric parameters (Å, °)

**Table:**

N1—C1	1.324 (3)	C5—H5	0.9500
N1—C9	1.387 (3)	C6—C7	1.410 (4)
N1—C10	1.486 (3)	C6—H6	0.9500
C1—C2	1.409 (3)	C7—C8	1.417 (4)
C1—H1	0.9500	C8—C9	1.354 (4)
C2—C3	1.416 (4)	C8—H8	0.9500
C2—C7	1.418 (4)	C9—H9	0.9500
C3—C4	1.374 (4)	C10—C10^i^	1.521 (5)
C3—H3	0.9500	C10—H10A	0.9900
C4—C5	1.408 (4)	C10—H10B	0.9900
C4—H4	0.9500	O1—H1A	0.90 (4)
C5—C6	1.364 (4)	O1—H1B	0.81 (5)
			
C1—N1—C9	121.6 (2)	C5—C6—H6	120.1
C1—N1—C10	120.1 (2)	C7—C6—H6	120.1
C9—N1—C10	118.3 (2)	C6—C7—C8	123.4 (3)
N1—C1—C2	120.9 (2)	C6—C7—C2	118.5 (3)
N1—C1—H1	119.5	C8—C7—C2	118.1 (2)
C2—C1—H1	119.5	C9—C8—C7	120.6 (2)
C1—C2—C3	120.5 (2)	C9—C8—H8	119.7
C1—C2—C7	118.6 (2)	C7—C8—H8	119.7
C3—C2—C7	120.9 (2)	C8—C9—N1	120.1 (3)
C4—C3—C2	118.9 (3)	C8—C9—H9	119.9
C4—C3—H3	120.6	N1—C9—H9	119.9
C2—C3—H3	120.6	N1—C10—C10^i^	109.4 (3)
C3—C4—C5	120.1 (3)	N1—C10—H10A	109.8
C3—C4—H4	119.9	C10^i^—C10—H10A	109.8
C5—C4—H4	119.9	N1—C10—H10B	109.8
C6—C5—C4	121.8 (3)	C10^i^—C10—H10B	109.8
C6—C5—H5	119.1	H10A—C10—H10B	108.2
C4—C5—H5	119.1	H1A—O1—H1B	99 (4)
C5—C6—C7	119.8 (3)		
			
C9—N1—C1—C2	0.0 (4)	C1—C2—C7—C6	−178.8 (2)
C10—N1—C1—C2	178.9 (2)	C3—C2—C7—C6	0.5 (4)
N1—C1—C2—C3	179.6 (3)	C1—C2—C7—C8	1.3 (4)
N1—C1—C2—C7	−1.0 (4)	C3—C2—C7—C8	−179.3 (3)
C1—C2—C3—C4	179.4 (3)	C6—C7—C8—C9	179.4 (3)
C7—C2—C3—C4	0.0 (4)	C2—C7—C8—C9	−0.7 (4)
C2—C3—C4—C5	−1.0 (4)	C7—C8—C9—N1	−0.2 (4)
C3—C4—C5—C6	1.4 (5)	C1—N1—C9—C8	0.6 (4)
C4—C5—C6—C7	−0.9 (4)	C10—N1—C9—C8	−178.2 (3)
C5—C6—C7—C8	179.7 (3)	C1—N1—C10—C10^i^	96.5 (3)
C5—C6—C7—C2	−0.1 (4)	C9—N1—C10—C10^i^	−84.7 (4)

Symmetry codes: (i) −*x*+1, −*y*+2, −*z*+2.

### Hydrogen-bond geometry (Å, °)

**Table:**

*D*—H···*A*	*D*—H	H···*A*	*D*···*A*	*D*—H···*A*
O1—H1A···Br1^ii^	0.90 (4)	2.41 (4)	3.308 (3)	176 (3)
O1—H1B···Br1^iii^	0.81 (5)	2.51 (5)	3.313 (3)	178 (5)
C1—H1···Br1^iv^	0.95	2.84	3.593 (3)	137
C9—H9···Br1^v^	0.95	2.81	3.691 (3)	154
C10—H10B···Br1^v^	0.99	2.87	3.683 (3)	140
C3—H3···O1^vi^	0.95	2.57	3.396 (4)	145
C4—H4···O1^vii^	0.95	2.54	3.380 (4)	147
C10—H10A···O1^iv^	0.99	2.27	3.214 (4)	158

Symmetry codes: (ii) *x*+1, *y*, *z*; (iii) −*x*+1, −*y*+1, −*z*; (iv) *x*, *y*+1, *z*+1; (v) −*x*+1, −*y*+1, −*z*+1; (vi) −*x*+1, −*y*+2, −*z*+1; (vii) *x*−1, *y*+1, *z*.
